# Pan-cancer analysis identifies FKBP10 as a regulator of tumor immunosuppression and therapeutic response

**DOI:** 10.1016/j.tranon.2026.102749

**Published:** 2026-04-03

**Authors:** Yan Li, Jian Chen, Siyan Li, Gang Tu, Lingfeng Tang

**Affiliations:** a Department of Breast and Thyroid Surgery, Chongqing Key Laboratory of Molecular Oncology and Epigenetics, The First Affiliated Hospital of Chongqing Medical University, Chongqing, 400016, China; b Department of Breast and Thyroid Surgery, The Second People's Hospital of Yibin, 644000, Sichuan, China

**Keywords:** FKBP10, Tumor cell fate, Programmed cell death, Therapeutic response, Pan-cancer

## Abstract

**Background:**

Tumor immune regulation depends on interactions between intrinsic molecular mechanisms, tumor cell metabolic states, and the immune microenvironment. The ER‑localized isomerase FK506‑binding protein 10 (FKBP10) is essential for extracellular matrix homeostasis, but its systemic role in cancer immune regulation and therapeutic response remains unclear.

**Methods:**

A pan‑cancer analysis of 33 TCGA tumor types integrated copy number variation, DNA methylation, and mutation data to elucidate FKBP10 regulation. ESTIMATE, ImmuCellAI, and TIMER2 assessed immune infiltration and immunosuppressive phenotypes, and pathway enrichment identified FKBP10‑related metabolic reprogramming, ECM remodeling, EMT, and programmed cell death signals. Therapeutic response was evaluated using breast cancer *in vitro* assays and GDSC drug sensitivity data.

**Results:**

FKBP10 was significantly upregulated in multiple cancers, especially breast and renal carcinoma, and strongly associated with poor prognosis. Its high expression was mainly driven by promoter hypomethylation and copy number amplification. FKBP10‑high tumors showed enhanced ECM remodeling, metabolic reprogramming, and apoptosis resistance, accompanied by an immunosuppressive microenvironment with reduced cytotoxic T cells and enrichment of myeloid‑derived suppressor cells and cancer‑associated fibroblasts. High FKBP10 expression also correlated with decreased sensitivity to several chemotherapeutic agents. In vitro experiments confirmed that FKBP10 promotes tumor cell proliferation and clonogenicity.

**Conclusions:**

FKBP10 is closely linked to tumor progression and treatment response by contributing to metabolic reprogramming, immunosuppressive microenvironment modulation, and programmed cell death regulation, supporting its potential as a biomarker and therapeutic target for predicting prognosis, treatment response, and immunotherapy outcomes.

## Highlights

• Pan-cancer analysis links FKBP10 overexpression to poor prognosis in breast/renal cancers.
• FKBP10 drives immunosuppressive TME via hypomethylation, reduced T-cells and increased MDSCs/CAFs.
• FKBP10 confers chemoresistance and promotes tumor proliferation in functional validation.

## Graphical abstract

**Figure:**
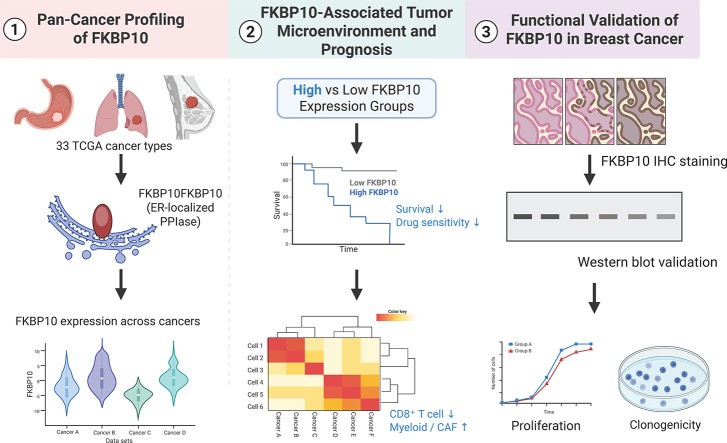

## Introduction

As a primary cause of global mortality, cancer represents a substantial public health issue. Despite significant progress in standard treatments like chemotherapy, radiotherapy, and surgery, along with the advent of novel approaches such as immunotherapy and targeted therapies, numerous cancers remain refractory to therapy. These malignancies are characterized by frequent relapse and metastatic dissemination [1]. Consequently, an urgent requirement exists for the discovery of new biomarkers and therapeutic avenues to enhance early detection, refine prognostic risk assessment, and augment the effectiveness of treatments for diverse cancer types [2].

The FK506-binding protein 10 (FKBP10) is an endoplasmic reticulum–resident peptidyl-prolyl *cis*–trans isomerase belonging to the FKBP family and has been implicated in collagen organization, protein folding, and cellular stress responses [3]. FKBP10 was initially studied in the context of rare genetic disorders such as Bruck syndrome and osteogenesis imperfecta, where mutations in this gene are associated with severe skeletal abnormalities [[4], [5], [6]]. However, the involvement of FKBP10 in cancer biology has not been fully elucidated and has only recently been explored. Recent studies suggest that FKBP10 expression is altered in several malignancies and may contribute to tumor progression through multiple mechanisms. Notably, FKBP10 has been shown to facilitate renal cell carcinoma progression by promoting LDHA phosphorylation and influencing HIFα-related immune regulation [7]. It is also known to enhance glioma growth through the AKT-CREB-PCNA pathway [8] and enabling muscle invasion in bladder cancer through disruption of lamin A [9]. Furthermore, its involvement has been suggested in the prognosis and chemotherapeutic response of breast cancer [10], while its suppression has been shown to mitigate fibrotic diseases like gluteal muscle contracture [11]. The clinical significance of FKBP10 is also suggested by its subcellular expression patterns in colorectal cancer [12], and its prognostic utility has been established in both lung adenocarcinoma [13] and clear cell renal cell carcinoma [14].

The majority of prior research, however, has concentrated on individual cancer types, leaving a comprehensive pan-cancer investigation of FKBP10 unperformed. Consequently, a systematic understanding of its functions related to tumor immunity, epigenetic control, genomic instability, and the tumor microenvironment is deficient. Moreover, although discrete studies point towards oncogenic roles, the broad applicability of FKBP10 as a biomarker and its connection to therapeutic responses across a spectrum of cancers remain unexplored.

These knowledge gaps can be filled thanks to the availability of large genomic and transcriptomic datasets, such as those from The Cancer Genome Atlas (TCGA) and the Genotype-Tissue Expression (GTEx) project, as well as advanced bioinformatic techniques. The objective of the current research is to perform an integrative multi-omics analysis to clarify the role of FKBP10 across multiple cancer types.

In this work, we methodically examined the expression of FKBP10 in a range of normal and cancerous tissues. We evaluated the prognostic significance of FKBP10 for different survival outcomes and investigated its correlation with immune cell infiltration, tumor microenvironment features, and gene mutations. Additionally, we examined drug sensitivity profiles and pathways linked to FKBP10, and we experimentally confirmed the functional role of FKBP10 in the development of breast cancer. These analyses' findings seek to identify FKBP10 as a crucial element in carcinogenesis and a possible target for treatment in a variety of malignancies.

## Materials and methods

### Source of the data

The UCSC Xena platform (https://xenabrowser.net/datapages/; accessed September 2023) was used to aggregate RNA-seq data and related clinical data from TCGA and GTEx. To mitigate technical batch effects and ensure cross-cohort comparability, we utilized the UCSC Toil RNA-seq Recompute dataset, which computationally harmonized raw RNA-seq data using a uniform bioinformatics pipeline (STAR, RSEM, and Kallisto). Gene expression levels were quantified as log2(TPM+0.001) (Transcripts Per Million). The cBioPortal database (https://www.cbioportal.org/) provided the DNA copy number and methylation information. Genes expressed in less than half of the samples were eliminated prior to additional analysis, and sample identifiers were standardized across datasets.

### Pan-cancer analysis

Expression differences across tumor types were visualized and compared using customized scripts built on the ggplot2 framework. To determine the statistical correlations between FKBP10 and established immunotherapy biomarkers in a pan-cancer context—such as immune cell infiltration, immune score, and other recognized immune-related genes—Spearman rank analysis was conducted. The cBioPortal tool (http://www.cbioportal.org/) will be used to evaluate the mutation patterns of the FKBP10 gene in different types of cancer.

### Drug sensitivity analysis

Using the oncoPredict R package (v0.2), bulk RNA-seq expression profiles were integrated with drug response–related genomic data from the GDSC2 (Genomics of Drug Sensitivity in Cancer) database to generate in silico predicted drug responses via default ridge regression models. Patients were stratified into high- and low-FKBP10 expression groups based on the median expression value. For each agent, sensitivity was quantified by estimating the corresponding half-maximal inhibitory concentration (IC50). Differences in drug response between groups were subsequently evaluated using the Wilcoxon rank-sum test, followed by Benjamini-Hochberg (BH) False Discovery Rate (FDR) adjustment to correct for multiple testing.

### Prognostic analysis

The R packages survival and survminer were used to perform Kaplan-Meier survival analysis. Patients in each cancer cohort were stratified into high- and low-FKBP10 expression groups based on the median expression cutoff. Univariate Cox regression was employed to evaluate the effect of FKBP10 expression on overall survival (OS), disease-specific survival (DSS), progression-free interval (PFI), and disease-free interval (DFI), with results visualized using the forestplot package. Furthermore, multivariable Cox proportional hazards models, adjusting for available clinical covariates (such as age, gender, and tumor stage), were applied to ascertain whether FKBP10 serves as an independent prognostic factor. Hazard ratios (HR) and their 95% confidence intervals (CI) were calculated.

### Genomic alteration and mutational burden analyses

The frequencies of genetic alterations in all cancer types, including somatic cell mutations, copy number variations, and deep deletions, were examined using the cBioPortal cancer type summary module. Copy number variation (CNV) profiles were obtained from the TCGA, and associations between different patient subgroups were assessed using Spearman sequence correlation analysis.

### FKBP10 DNA methylation analyses

DNA methylation profiles were downloaded from cBioPortal and analyzed to explore the correlation between FKBP10 promoter methylation and mRNA expression. Spearman’s rank correlation coefficients were calculated using the ggpubr package in R to evaluate this association across multiple cancer types.

### Functional enrichment analyses

To elucidate the biological processes associated with FKBP10, pathway activity and functional enrichment analyses were carried out using transcriptomic profiles. Gene set variation analysis (GSVA) was performed based on the GSVA package in R to estimate the activity scores of biological pathways in each sample based on the signature gene set (h.all.v7.4.symbols.gmt) which obtained from the Molecular Signature Database (MSigDB). Associations between FKBP10 expression levels and pathway activity scores were then examined in the TCGA-BRCA cohort using Spearman rank correlation analysis. Furthermore, the top 100 genes exhibiting expression patterns most similar to FKBP10 were identified via the Similar Gene Detection module of GEPIA2. Gene annotations from the org.Hs.eg.db database were then used to perform gene ontology (GO) enrichment analysis on these co-expressed genes using the R clusterProfiler package. A GO term was deemed statistically significant if its P-value, adjusted for false discovery rate (FDR), was less than 0.05. Furthermore, the global pathway differences between samples with high and low FKBP10 expression were investigated using Gene Set Enrichment Analysis (GSEA). The Hallmark gene set from MSigDB was employed to analyze outcomes after stratifying patients into high-low FKBP10 expression groups based on the mean expression level. Furthermore, the EMT1, EMT2, and Pan-fibroblast TGF-β response signatures (Pan.F.TBRS), which are well-established transcriptomic markers for stromal activation, extracellular matrix remodeling, and immune evasion, were evaluated based on gene sets curated by Mariathasan et al [15].

### Immunohistochemistry (IHC)

Breast cancer tissue samples that had been paraffin-embedded and formalin-fixed, as well as nearby normal tissue, were cut into thin pieces that were 3–4 µm thick, dried, and incubated for three hours at 60 °C. After that, the paraffin was removed using xylene, immersed in increasingly concentrated ethanol to hydrate it, and then rinsed with phosphate buffer solution (PBS). Heat under pressure in citrate buffer (pH 6.0) was used to remove the antigen for ten minutes at 92 °C, after which it was cooled to room temperature. 3% hydrogen peroxide was used to inhibit peroxidase activity in the tissue for ten minutes, and an hour of room temperature incubation prevented non-specific antibodies from attaching to normal goat serum (BOSTER, China).

The sections were then treated with a diluted primary antibody targeting FKBP10 (Proteintech, China) for an entire night at 4 °C in accordance with the manufacturer's instructions. Following a thorough PBS cleaning, samples were subjected to a species-matched HRP-conjugated secondary antibody (BOSTER, China) for half an hour at room temperature. DAB color-forming reagent (ZSGB-BIO, China) was used for signal analysis, and hematoxylin (Solarbio, China) was used for nuclear counterstaining. After that, the slides underwent dehydration, xylene clearing, and neutral resin mounting.Brown cytoplasmic signals were indicative of positive immunostaining. Five randomly chosen fields per section were photographed at × 400 magnification using a light microscope.

### Construction of FKBP10 overexpression and knockdown cell lines

The full-length human FKBP10 genetic sequence was amplified by PCR from a cDNA library using Phanta Super-Fidelity DNA Polymerase (Vazyme, China) and inserted into the LV-ECMV-PURO viral vector via the *Bam*HI and EcoRI restriction cut-off sites (New England Biolabs, USA). For FKBP10 knockdown, short hairpin RNA (shRNA) target sequences were designed with the BLOCK-iT RNAi Designer (Invitrogen, USA). The annealed shRNA oligonucleotides were ligated into the LV-shRNA-PURO vector. The target sequences were as follows: shFKBP10–1, CTACCACTACAACGGCACTTT; shFKBP10–2, GCGGCACTTATGACACCTACG.

Lentivirus vectors were created by co-transfecting the FKBP10 overexpression construct or shRNA plasmid with the psPAX2 and pMD2.G packaging vectors (Addgene, USA) into Lenti-X 293T cells (Takara, Japan) using Lipofectamine 3000 (Thermo Fisher Scientific, USA) following the manufacturer’s protocol. 48 h after transfection, the viral supernatant was harvested, cleared by centrifugation to remove cellular debris, and passed through a 0.45 µm pore filter.

Human breast cancer cell lines MCF7 and T47D were routinely maintained in Dulbecco’s Modified Eagle Medium (DMEM, Gibco, USA) supplemented with 10% fetal bovine serum (FBS, Gibco, USA) and 1% penicillin-streptomycin at 37 °C in a humidified 5% CO₂ atmosphere. MCF7 and T47D cells were subsequently cultured in six-well plates and transfected with a virus lentivirus solution prepared using 8 μg/ml Polybrene (Beyotime, China). The 24 h infection, the inoculum was removed and replaced with complete culture medium, and cells were selected using puromycin to remove all uninfected control cells. Successfully transfected cells with FKBP10 overexpression or shRNA inhibition were expanded and verified by RT-qPCR and Western blotting for further analysis.

### Real-time quantitative PCR (RT-qPCR)

The RT Master Mix for qPCR II (MedChemExpress, USA) was used to synthesize complementary DNA (cDNA) from one microgram of total RNA. The CFX96 Real-Time qPCR Detection System (Bio-Rad, USA) and SYBR Green qPCR Master Mix (MedChemExpress, USA) were used for quantitative real-time qPCR analysis. The primer sequences used were as follows: GAPDH forward, GGAGCGAGATCCCTCCAAAAT; GAPDH reverse, GGCTGTTGTCATACTTCTCAGG; FKBP10 forward, GCCTTCTTCACCATCTTCCT; and FKBP10 reverse, GTCCAGGTCCTCATCTTGGT. The 2⁻ΔΔCt method was utilized to calculate relative mRNA transcript levels using GAPDH as the endogenous reference gene. Each qPCR reaction was performed three times.

### Western blot

The cells were thawed on ice in RIPA buffer (Biyotime, China) supplemented with protease and phosphatase inhibitors after being cleaned with ice-cold phosphate-buffered saline (PBS). After a brief sonication, the resulting lysate was centrifuged at 12,000 × *g* for 7 min at 4 °C. A BCA protein assay kit (Biyotime, China) was applied to measure the total protein amount. Equal volumes of protein were mixed with loading buffer, boiled for ten minutes to denature it, separated using SDS-PAGE, followed by transfer onto PVDF membranes.

Primary antibodies against FKBP10 and GAPDH (Proteintech, China) were incubated at room temperature for 2 h, followed by blocking the membrane with 5% non-fat milk in TBST overnight at 4 °C. After washing, the membrane was exposed to HRP-linked secondary antibodies (Beyotime, China) for 2 h at room temperature. Enhanced chemiluminescence (ECL) reagent (NCM Biotech, China) was used to identify immunoreactive bands, and a Bio-Rad imaging system was used to visualize them. ImageJ software was used to measure band intensities, and the protein expression levels of FKBP10 were normalized against GAPDH.

### CCK-8 cell viability assay

The CCK-8 cell count kit (CCK-8; MedChemExpress, USA) was used to measure cell proliferation. In short, 96-well plates with 200 μL of DMEM (Gibco, USA) supplemented with 10% FBS were seeded with 5 × 10³ cells per well. Following overnight fixation, cells were cultured for the predetermined amount of time after being treated with the indicated medications or the control vehicle. The plates were incubated for one to two hours at 37 °C after 200 μL of CCK-8 working solution (10% CCK-8 diluted in DMEM) was added to the culture medium at each time point. A microplate reader (Thermo Fisher Scientific, USA) was applied to record absorbance at 450 nm. At least three biological replicates were used for each measurement.

### Colony formation assay

Trypsinization was used to extract and quantify cells in the exponential growth phase for colony formation assays. Each well of a 6-well plate was seeded with 1000 cells, and the cells were cultivated in full culture medium for 7–14 days, with medium replenishment as necessary. Following the appearance of clear colonies, the cells were gently cleaned with PBS, then fixed for 15 min with 4% paraformaldehyde, and stained. Staining was performed using 0.1% crystal violet for at least half an hour (Beyotime, China). Colonies with 50 or more cells were counted under a light microscope following washing and air drying. Every experiment was carried out in triplicate.

### Statistical analysis

All statistical computations and data analyses were performed in R software (version 4.3.1). Key R packages included survival (v3.5–5), GSVA (v1.48.0), clusterProfiler (v4.8.1), and maftools (v2.16.0). To account for multiple testing in high-dimensional pan-cancer analyses (e.g., immune correlations, drug sensitivity, and pathway enrichments), P-values were adjusted using the Benjamini-Hochberg (BH) method to control the False Discovery Rate (FDR). Statistical significance was defined as an adjusted P-value (FDR) < 0.05, unless otherwise specified. To evaluate the differences between two independent groups, the Wilcoxon rank-sum test was utilized. The survival rates were evaluated using the Kaplan-Meier test, and correlations between variables were examined with the Spearman rank correlation coefficient.

## Result

### Pan-Cancer evaluations of FKBP10 expression

**Fig. 1:**
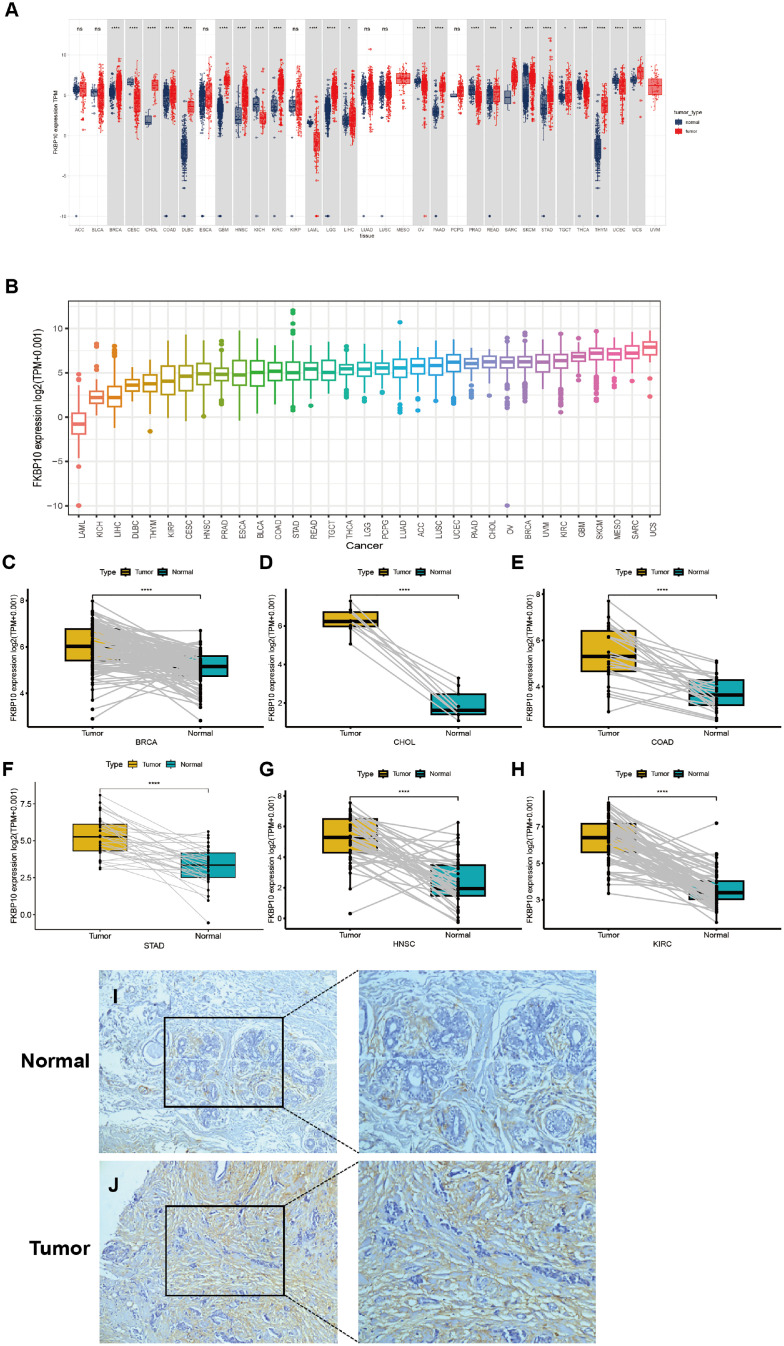
Pan-cancer expression pattern of FKBP10. (A) Expression levels of FKBP10 in cancer tissues versus corresponding healthy normal tissues across 33 tumor types. (B) Relative FKBP10 expression among different tumor types. (C–H) Paired comparisons of FKBP10 expression between tumor and adjacent normal tissues in selected cancers. (I–J) Immunohistochemical staining of FKBP10 in normal breast tissue and breast cancer tissue.

A systematic pan-cancer investigation of FKBP10 mRNA expression was initially undertaken using the TCGA and GTEx databases. This examination identified differential expression across 33 cancer types (Fig. 1A). Compared to adjacent normal samples, FKBP10 expression was elevated in BRCA, CHOL, COAD, DLBC, GBM and additional tumor types. Conversely, the expression of FKBP10 was reduced in CESC, KICH, LAML, OV, PRAD, THCA, and UCEC (Fig. 1A). Specifically, FKBP10 expression was remarkably elevated in cancerous tissues versus healthy normal samples in BRCA, CHOL, COAD, STAD, HNSC, and KIRC (Fig. 1C—H). The relative expression levels of FKBP10 among different cancer types are depicted in Fig. 1B. Tumors derived from GBM, SKCM, MESO, SARC, and UCS demonstrated higher FKBP10 expression (Fig. 1B). For the experimental validation of these findings, IHC staining of FKBP10 proteins was carried out on breast cancer sections. The outcomes suggested that FKBP10 expression was heightened in cancerous tissue relative to normal breast tissue, as reflected by an increase in the mean optical density (Fig. 1I-J). These findings highlight a widespread dysregulation pattern of FKBP10 across human cancers.

### Pan-cancer evaluation of FKBP10′s complex prognostic significance

**Fig. 2:**
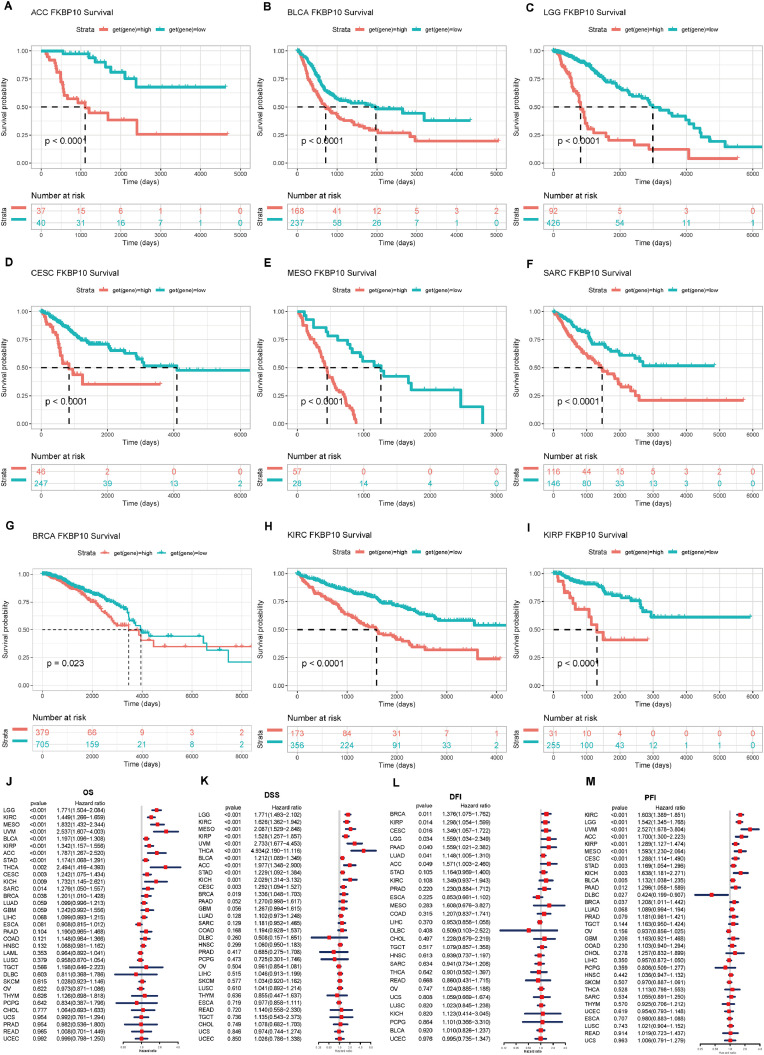
The prognostic value of FKBP10 in various cancers (A–I). The Kaplan-Meier survival graph shows the difference in overall survival (OS) between groups with high and low FKBP10 expression in ACC, BLCA, LGG, CESC, MESO, SARC, BRCA, KIRC, and KIRP (J–M). The Forest plot obtained from univariate Cox regression analysis shows the relationship between FKBP10 expression and OS, DSS, DFI, and PFI in 33 cancers.

Using the complete cancer dataset, the relationship between FKBP10 expression and patient prognosis was examined. OS, DSS, DFI, and PFI were among the survival indicators. Higher FKBP10 expression was significantly linked to a poor prognosis in ACC, BLCA, LGG, CESC, MESO, SARC, BRCA, KIRC, and KIRP, according to Kaplan-Meier analysis in 33 cancer types (Fig. 2A-I). FKBP10 expression was substantially linked to OS in a number of cancer types, including STAD, KIRC, UVM, BLCA, KIRP, and BRCA, according to Cox regression analysis in 33 cancer types (Fig. 2J-M). Higher FKBP10 expression in these cancers was significantly linked to a worse prognosis (Fig. 2J).

Additionally, the relationship between DSS and FKBP10 expression was evaluated. In a number of cancer types, including LGG, KIRC, MESO, KIRP, UVM, THCA, BLCA, ACC, STAD, KICH, CESC, and BRCA, expression of FKBP10 was found to affect DSS (Fig. 2K). Poor DSS was associated with elevated FKBP10 expression in patients with these cancers (Fig. 2K). FKBP10 expression affected patients with BRCA, KIRP, CESC, LGG, PAAD, LUAD, and ACC, according to a DFI analysis (Fig. 2L). In terms of PFI, the Cox regression analysis showed that FKBP10 expression had an impact on a number of cancers, such as KIRC, LGG, KIRP, CESC, BLCA, PAAD, and BRCA (Fig. 2M). Importantly, multivariable Cox regression confirmed that FKBP10 remained an independent prognostic indicator for OS in several tumor types, including BRCA and KIRC, even after adjusting for clinical covariates such as age and stage.

The consistent associations between FKBP10 expression and multiple survival endpoints further support its prognostic relevance in cancer.

### Mutational analysis of FKBP10 in various tumors

**Fig. 3:**
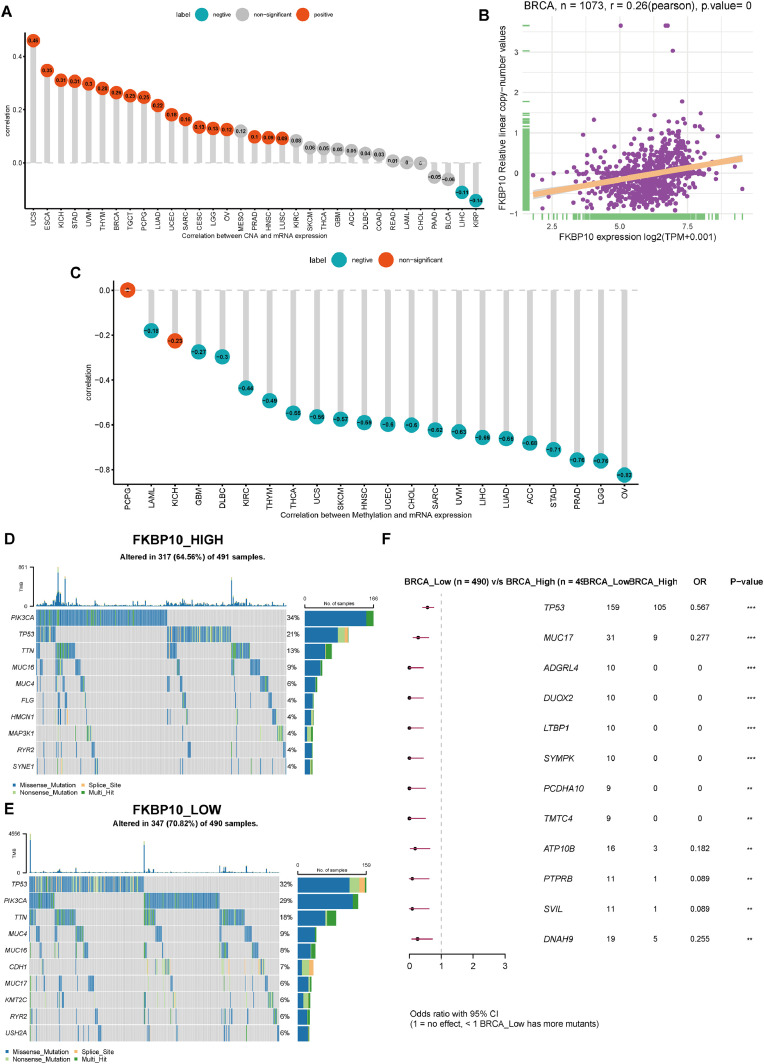
Genomic and epigenetic alterations associated with FKBP10. (A) Correlation between copy number variations (CNVs) and FKBP10 expression across cancers. (B) Association between FKBP10 expression and copy number levels in BRCA. (C) Correlation between FKBP10 promoter methylation and mRNA expression across cancers. (D–E) Mutation profiles of the top 10 highest-frequency mutation-enriched genes in BRCA groups with high-low FKBP10 expression. (F) Differential mutation landscape in BRCA between groups with high or low FKBP10 expression.

A frequent characteristic of cancer is heightened genomic instability; nevertheless, the manner in which genomic alterations correspond with FKBP10 expression across numerous cancers has yet to be elucidated. To investigate this, the associations between FKBP10 expression levels and both CNVs and DNA methylation were explored. Subsequent CNV correlation analyses showed significant correlations in 20 out of 33 cancer types, most notably in UCS, ESCA, KICH, STAD, UVM, THYM, and BRCA (Fig. 3A). A positive relationship between FKBP10 expression and CNV in BRCA was also observed, as depicted in Fig. 3B The analysis of the connection between FKBP10 expression and DNA methylation suggested that methylation led to a significant suppression of FKBP10 expression in OV, LGG, PRAD, STAD, and ACC (Fig. 3C).

The UCSC Xena database was used to obtain gene mutation information for every type of tumor. Next, we used the "maftools" R package to visualize gene mutations in groups with high and low FKBP10 gene expression. In the high FKBP10 expression group, the 10 most commonly mutated genes were PIK3CA, TP53, TTN, MUC16, MUC4, FLG, HMCN1, MAP3K1, RYR2, and SYNE1. TP53, PIK3CA, TTN, MUC4, MUC16, CDH1, MUC17, KMT2C, RYR2, and USH2A constituted the top 10 genes exhibiting the highest mutation rates in the low FKBP10 group (Fig. 3D-E). Additionally, we used risk ratio analysis to evaluate the variations in BRCA gene mutation frequencies between groups with high and low FKBP10 expression. Several genes, including TP53, MUC17, ADGRL4, DUOX2, LTBP1, SYMPK, PCDHA10, TMTC4, ATP10B, PTPRB, SVIL, and DNAH9, showed significantly different mutation rates between the two subgroups (Fig. 3F).

These findings imply that both genetic and epigenetic events are important contributors to the abnormal expression of FKBP10 in pan-cancer.

### Tumor microenvironment analysis for FKBP10 in pan-cancer and BRCA

**Fig. 4:**
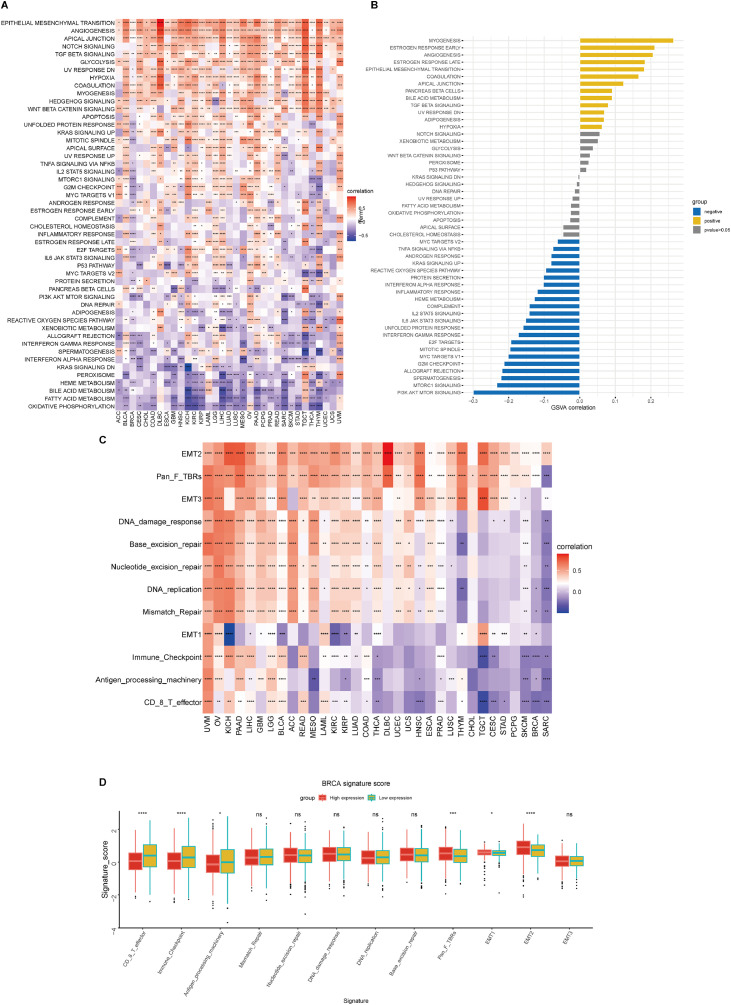
GSVA and tumor microenvironment analysis (A) GSVA showing the pan-cancer correlation of FKBP10 expression with defined biological pathways. (B) GSVA concentration scores of specific biological pathways in TCGA-BRCA samples. (C) Correlation between FKBP10 expression and TME scores in all cancer types. (D) Comparison of TME-associated specific biological pathways between groups with high and low FKBP10 expression in BRCA.

Abbreviations: EMT1/EMT2, epithelial-mesenchymal transition signatures; Pan.F.TBRS, Pan-fibroblast TGF-β response signature (derived from Mariathasan et al., 2018).

To examine the potential role of FKBP10 in tumor biology, we employed GSVA to evaluate the association between FKBP10 expression and significant signaling pathways in various cancer types. FKBP10 expression was found to be positively associated with a number of tumor-promoting programs, such as angiogenesis, apical junctions, NOTCH signaling, and the epithelial-to-mesenchymal transition. However, across a variety of tumor types, FKBP10 expression was negatively correlated with metabolic pathways like oxidative phosphorylation (Fig. 4A). Furthermore, within the TCGA-BRCA cohort, FKBP10 gene expression was positively correlated with pathway scores such as myogenesis, early estrogen response, angiogenesis, epithelial-to-mesenchymal transition, and apical junction. Conversely, negative correlations were found between FKBP10 gene expression and scores of pi3k-akt-mtor signaling, mtorc1 signaling, spermatogenesis, graft rejection, and g2m monitoring pathways (Fig. 4B).

TME analysis revealed a positive correlation between FKBP10 and TME in several cancer types, including UVM, OV, KICH, PAAD, LIHC, GBM, LGG, and BLCA. Meanwhile, there was a negative correlation between FKBP10 and TME in PCPG, SKCM, BRCA, and SARC (Fig. 4C). Another significant finding was the difference in TME expression between the groups with high FKBP10 and low BRCA expression. More importantly, The analysis further demonstrated a significant upregulation of EMT1, EMT2, and Pan.F.TBRS signatures in the high-FKBP10 expression group (Fig. 4D).

Together, these data underscore a strong association between FKBP10 expression and key oncogenic pathways and tumor microenvironment characteristics.

### The connection between immune cell infiltration and FKBP10 expression

**Fig. 5:**
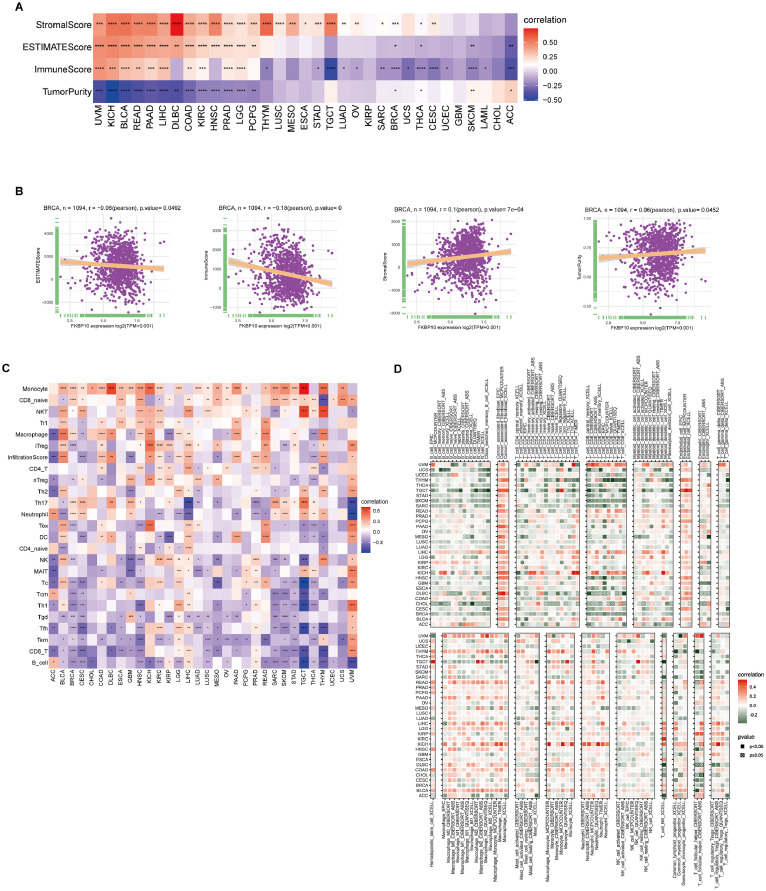
FKBP10 expression correlates with immune infiltration. (A) Correlation matrix showing associations between FKBP10 expression and TME-related metrics. (B) Scatter plots illustrating correlations between FKBP10 and TME-related scores in BRCA. (C) Heatmap showing the associations of FKBP10 expression with immune cell subsets based on ImmuCellAI database. (D) Associations between FKBP10 expression and immune infiltration levels estimated by TIMER2 across cancers.

Cancer cell behavior is significantly influenced by the surrounding cellular environment within the TME. In this work, we methodically examined the connection between FKBP10 expression and a number of TME-related metrics. Across multiple cancer types, FKBP10 expression showed an overall inverse association with tumor purity, most prominently in UVM, KICH, BLCA, READ, and LIHC (Fig. 5A). FKBP10 expression in BRCA had a weak positive correlation with tumor purity and stromal score, but a weak negative correlation with immune score and ESTIMATE score (Fig. 5B). FKBP10 expression and immune scores were found to be positively correlated in UVM, KICH, BLCA, LIHC, and COAD. Based on data from the ImmuCellAI database, a high positive correlation was observed between FKBP10 and several immune cell types, including monocytes, CD8 naive, and NKT cells. FKBP10 was observed to be negatively related to CD8+*T*, Tem, Tfh, Tgd, and Th1 cells (Fig. 5C). Next, we assessed possible correlations between changes in immune cell infiltration and FKBP10 gene expression using the TIMER2 algorithm. Across multiple cancer types, FKBP10 levels were inversely correlated with lymphocyte populations, including B and T cells. In contrast, the quantity of endothelial cells, neutrophils, macrophages, and other bone marrow-derived cells was positively connected with increased FKBP10 expression. Additionally, in some tumor types, increased infiltration of cancer-associated fibroblasts was correlated with increased expression of FKBP10 (Fig. 5D). In summary, the above findings indicate that FKBP10 was closely associated with immune system modifications and changes in the tumor microenvironment, which may promote tumor progression.

### Validation of FKBP10 co-expression networks in BRCA

**Fig. 6:**
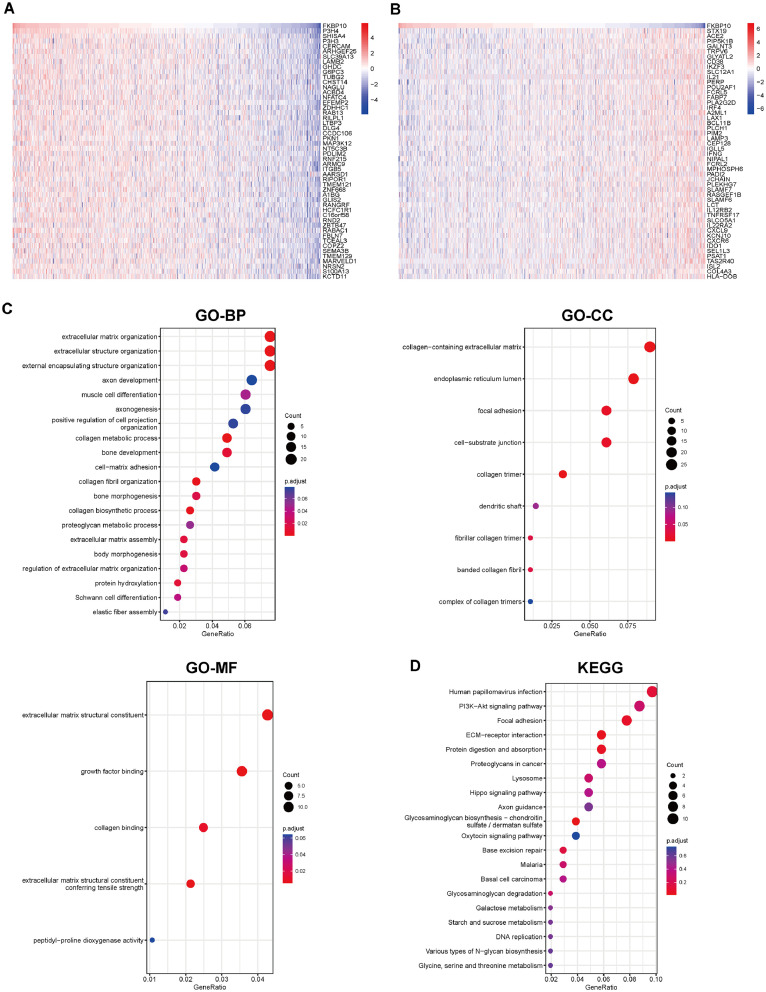
Co-expression network of FKBP10 and its functional enrichment analysis. (A–B) Heat chart showing the most positively and negatively associated genes with FKBP10 in the TCGA-BRCA database. (C) Gene Ontology (GO) enrichment analysis identifying biological processes (BP), cellular components (CC), and molecular functions (MF). (D) KEGG pathway enrichment analysis showing pathways significantly associated with FKBP10-related genes.

Given the pronounced pan-cancer heterogeneity of FKBP10, we selected Breast Invasive Carcinoma (BRCA) as a pre-specified, representative case study for deep molecular and functional validation. This selection was based on our aforementioned findings that FKBP10 is profoundly upregulated, strongly prognostic, and significantly associated with high mutation burdens and ECM remodeling specifically within the BRCA cohort. The preceding results established a significant association of FKBP10 with tumor prognosis and immunity. Consequently, a further verification of the KEGG pathways and GO terms linked to FKBP10 and its associated genes was performed in BRCA. Figs. 6A and B illustrate the top 50 genes showing either positive or negative associations with FKBP10 expression in BRCA. Based on these co-expressed genes, functional annotation analyses were performed using GO and KEGG databases (Fig. 6C-D). These genes were mainly linked to biological processes involving extracellular matrix organization and remodeling, collagen biosynthesis and assembly, and proteoglycan metabolism, according to GO enrichment analysis. The enriched genes were mainly found in the extracellular matrix containing collagen, focal adhesions, cell–matrix junctions, and the endoplasmic reticulum lumen. At the molecular function level, there was significant enrichment in function which related to ECM organization, growth factor interactions, and post-translational protein modifications. Additionally, KEGG pathway enrichment analysis showed that these FKBP10 related genes were primarily involved in pathways associated with tumor cell adhesion, extracellular matrix remodeling, and cancer-related signal transduction.

### FKBP10 correlates with drug sensitivity

**Fig. 7:**
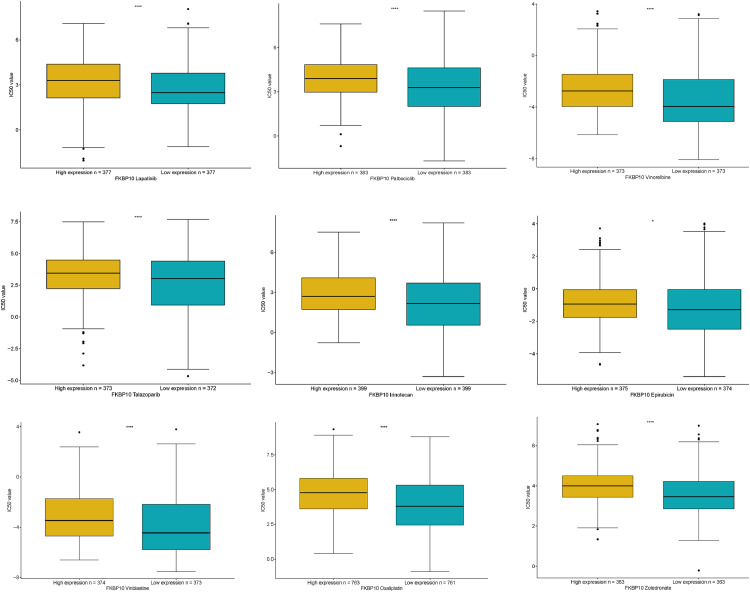
The relationship between FKBP10 expression and drug response. Boxplots comparing predicted IC50 values between high-low expression of FKBP10 groups for several anti-cancer drugs.

To further investigate how FKBP10 expression affects sensitivity to commonly used anti-cancer medications, a comparison of their IC50 values was made between high-low FKBP10 expression groups. As presented in Fig. 7, the in silico predicted IC50 values for several anti-cancer drugs, including lapatinib, palbociclib, vinorelbine, talazoparib, irinotecan, epirubicin, vinblastine, oxaliplatin, and zoledronate, were found to be significantly elevated (FDR < 0.05) in the high-FKBP10 group. This computational finding suggests that patients with lower FKBP10 expression may exhibit greater sensitivity to these chemotherapeutic agents. This finding suggests that patients with lower FKBP10 expression exhibit greater sensitivity to these chemotherapeutic agents.

### Functional validation of FKBP10 in breast cancer

**Fig. 8:**
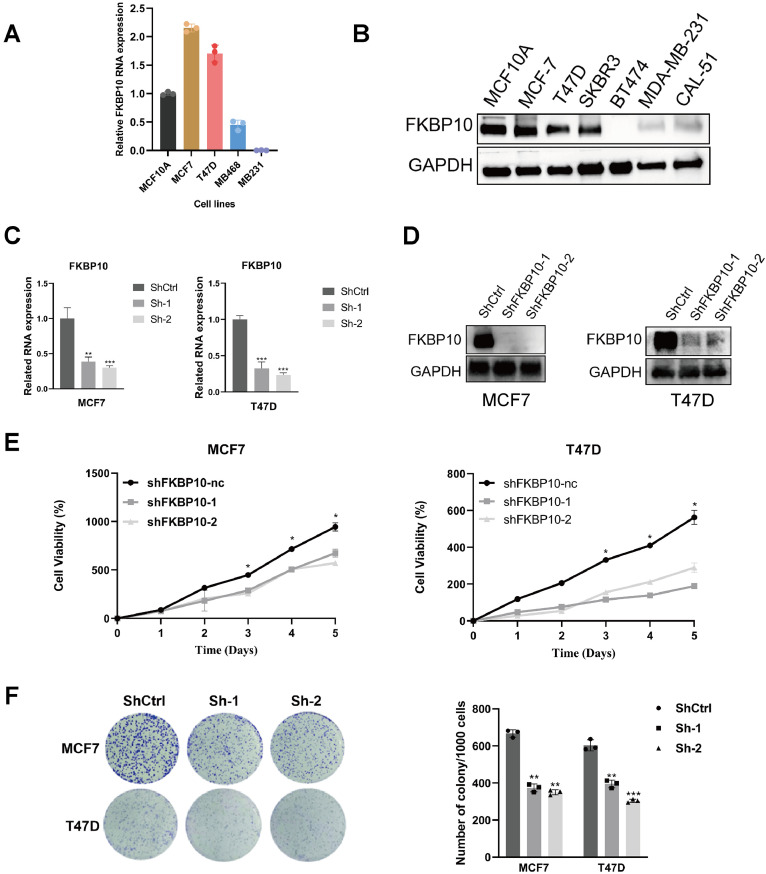
Functional validation of FKBP10 in breast cancer. (A) Relative FKBP10 mRNA expression in breast cell lines determined by qRT-PCR. (B) WB analysis of FKBP10 protein levels in breast cancer cell lines. (C) qRT-PCR verification of FKBP10 knockdown efficiency in MCF7 and T47D cells. (D) Western blot confirmation of FKBP10 knockdown efficiency. (E) CCK-8 assays showing reduced proliferation upon FKBP10 knockdown. (F) Colony formation assays showing decreased clonogenic capacity. Statistical differences were evaluated using the Wilcoxon rank-sum test followed by Benjamini-Hochberg FDR correction.

Quantitative RT–PCR and Western blot analyses were performed to characterize basal FKBP10 expression in breast epithelial and cancer cell lines (Fig. 8A,B). Based on their stable expression and experimental tractability, MCF7 and T47D were selected for subsequent functional assays.

To assess the loss-of-function effects of FKBP10, two independent shRNAs (shFKBP10–1 and shFKBP10–2) were introduced into MCF7 and T47D cells. Efficient knockdown of FKBP10 was confirmed by both qRT-PCR and Western blot (Fig. 8C–D). Functional assays revealed that downregulation of FKBP10 significantly reduced the proliferative capacity of breast cancer cells, as demonstrated by the CCK-8 assay, which showed a significant decrease in cell viability over time in both cell lines (Fig. 8E). In addition, colony formation assays revealed a substantial decrease in clonogenic ability following FKBP10 depletion (Fig. 8F), indicating a critical role for FKBP10 in sustaining long-term proliferative potential.

Conversely, FKBP10 overexpression was established in MCF7 and T47D cells to examine its functional significance in breast cancer cells. Both qRT-PCR and Western blot confirmed robust upregulation of FKBP10 following transfection with lentiviral overexpression vectors (Supplementary Figure 1A–B). Consistently, enforced expression of FKBP10 markedly enhanced cell viability and colony-forming ability in both cell lines (Supplementary Figure 1C–D), further confirming that FKBP10 positively regulates breast cancer cell proliferation and clonogenicity.

## Discussion

Cancer remains a serious global health problem, and the identification of a new biomarkers and molecular targets for therapy is essential to improve the patient outcomes [16,17]. For the first time, our research has presented a comprehensive pan-cancer overview of the oncogenic functions and clinical significance of FKBP10. FKBP10 is extensively expressed in a number of cancer types, according to multidimensional bioinformatics analysis and experimental validation. A poor prognosis, genomic changes (such as CNVs, mutation patterns, and DNA methylation changes), a changed tumor microenvironment, and decreased sensitivity to several chemotherapeutic agents are all closely associated with this upregulation.These findings suggest that FKBP10 is a key factor in cancer progression and a promising option for use as a biomarker and therapeutic target.

FKBP10 expression was found to be significantly higher in a number of cancers, including BRCA, CHOL, COAD, GBM, and LIHC, when compared to normal tissues. This observed upregulation is in line with its previously identified functions in stimulating tumor growth and metastasis in cancers such as glioma and clear cell renal cell carcinoma [[18], [19], [20]]. Significantly, in a number of aggressive cancers, such as ACC, LGG, KIRC, and BRCA, increased FKBP10 expression was linked to worse outcomes in terms of OS, DSS, PFI, and DFI. These results are consistent with earlier research linking FKBP10 to poor prognoses in gastric and renal cancers [21,22], highlighting its extensive oncogenic characteristics.

On a genomic scale, FKBP10 expression was intimately associated with copy number variations in cancers like UCS, ESCA, and BRCA, which suggests that copy number gains and amplifications could be a primary mechanism behind its overexpression. Furthermore, promoter hypermethylation and FKBP10 expression were found to be inversely correlated in OV, LGG, and PRAD, suggesting that epigenetic silencing may be a factor in FKBP10 dysregulation. Additionally, mutational analysis revealed that tumors with high FKBP10 expression had unique mutation profiles. For instance, in BRCA, frequent mutations in TP53 and PIK3CA co-occurred with FKBP10 overexpression, indicating possible synergistic interactions in promoting tumorigenesis. These results are in harmony with studies that emphasize FKBP10′s function in collagen cross-linking and extracellular matrix (ECM) remodeling [21,23,24], processes that are fundamental to cancer invasion and metastasis.

A particularly noteworthy finding of our investigation is the connection between FKBP10 and the tumor immune microenvironment. It was observed that FKBP10 expression had a negative correlation with tumor purity and was linked to immune suppression in multiple cancers, such as KICH, BLCA and READ. Elevated FKBP10 expression was linked to reduced infiltration of cytotoxic and helper T lymphocytes, accompanied by increased abundance of macrophages and cancer-associated fibroblasts. This finding suggests that FKBP10 might foster an immune-evasive TME, potentially through ECM remodeling and altered immune cell trafficking. Such insights are corroborated by recent research demonstrating that enzymes which modify collagen can affect T-cell exclusion and the response to immunotherapy [24,25]. Particularly, a recent multi-omics integration study identified FKBP10 as a crucial collagen-related biomarker driving intratumoral heterogeneity and profound ECM remodeling in hepatocellular carcinoma, which ultimately diminishes the therapeutic efficacy of immune checkpoint blockade (ICB) [26]. Moreover, FKBP10′s involvement in endoplasmic reticulum stress and protein folding [23,27,28] could further compromise antigen presentation and encourage immune tolerance. It is worth noting that minor discrepancies regarding CD8+ *T* cell correlations were observed between different deconvolution algorithms (e.g., negative correlation in ImmuCellAI versus positive correlation in TIMER2 for certain cancers). This discrepancy likely stems from algorithmic differences rather than biological contradictions: ImmuCellAI emphasizes the relative proportions of functional subsets, reflecting a phenotypic shift away from active cytotoxic T cells, whereas TIMER2 estimates overall lineage abundance. This highlights a methodological limitation intrinsic to bulk transcriptomic deconvolution, where functionally excluded T cells may still be computationally quantified alongside generalized leukocyte infiltration.

FKBP10 is linked to important carcinogenic processes like angiogenesis, ECM-receptor interaction, and epithelial-mesenchymal transition (EMT), according to analyses of pathway enrichment. Within BRCA, genes that were co-expressed with FKBP10 showed enrichment in collagen biosynthesis, extracellular matrix organization, and PI3K-Akt signaling-pathways recognized for their crucial roles in tumor progression and resistance to therapy [29,30]. These functional links are congruent with the established biochemical functions of FKBP10 in collagen maturation and stability [31,32], which are processes co-opted by cancer to facilitate invasion and metastatic dissemination.

Crucially, our study demonstrated that FKBP10 expression affects the response to chemotherapy. Patients exhibiting high levels of FKBP10 displayed greater resistance to a variety of drugs, such as lapatinib, palbociclib, vinorelbine and talazoparib. This indicates that FKBP10 may act as a predictive biomarker for drug sensitivity, thus providing a basis for combination therapies that target FKBP10 in tumors that are resistant to treatment. Our experimental validation in breast cancer cells confirmed that the knockdown of FKBP10 reduces clonogenicity and proliferation, which reinforces its functional significance in tumor growth and corroborates previous findings in other cancer types [33,34].

It is necessary to recognize, however, that uniform associations between FKBP10 and immune or molecular characteristics were not observed across all cancer types. For example, FKBP10 expression was lower in tumors than in normal tissue in PRAD and THCA, and its prognostic impact was not as strong. Such variations may be attributable to tissue-specific biological contexts or differences in the primary driver mutations, which underscores the importance of a cautious interpretation of pan-cancer findings.

Certain limitations of our research should be acknowledged. First, our pan-cancer transcriptomic analyses are fundamentally correlational; thus, causal relationships between FKBP10 and specific immune microenvironment alterations cannot be definitively established from bulk RNA-seq data alone. Second, estimates of immune cell infiltration were derived from algorithmic deconvolution, which is inherently susceptible to confounding by tumor purity—a critical factor in highly desmoplastic tumors like BRCA. Therefore, inverse correlations with immune cells should be interpreted with caution, as they may partially reflect the "immune-excluded" structural barriers shaped by CAFs rather than direct immune cell depletion. Third, our drug sensitivity findings are based on in silico predictions rather than direct clinical trial response data. Therefore, more *in vivo* lineage-tracing, spatial transcriptomic studies, and prospective clinical trials are needed to elucidate the precise mechanisms through which FKBP10 regulates chemoresistance and immune evasion in diverse tumor contexts.

In summary, our findings indicate that FKBP10 plays an important role in tumor development, immune regulation, and therapeutic response. Its consistently elevated expression and significant association with patient outcomes across multiple cancer types suggest that FKBP10 may serve as a useful diagnostic and prognostic biomarker. Moreover, these results provide a rationale for further exploration of FKBP10 as a potential therapeutic target, particularly in combination with existing immunotherapeutic or chemotherapeutic strategies. Future research should concentrate on clarifying the molecular mechanisms through which FKBP10 governs the TME and on validating its effectiveness in prospective clinical trials.

## Funding statement

This research was funded by 10.13039/100007452WU JIEPING MEDICAL FOUNDATION of Funder, grant number (No. 32067502025-21–18).

## Availability of data and materials

The data supporting the findings of this study are available from the corresponding author upon reasonable request.

## Ethics approval

The Declaration of Helsinki and national and international ethical guidelines were followed in the conduct of this study. The use of clinical materials was approved by the Ethics Committee of the First Affiliated Hospital of Chongqing Medical University (the approval number: K2023–514), and all patients gave their informed consent before the study began.

## CRediT authorship contribution statement

**Yan Li:** Methodology, Software, Validation, Formal analysis, Investigation, Resources, Data curation, Writing – original draft, Visualization. **Jian Chen:** Methodology, Software, Validation, Formal analysis, Investigation, Data curation, Writing – original draft, Visualization. **Siyan Li:** Software, Validation, Formal analysis, Resources, Data curation, Writing – original draft, Visualization. **Gang Tu:** Conceptualization, Validation, Writing – review & editing, Supervision, Project administration, Funding acquisition. **Lingfeng Tang:** Methodology, Software, Validation, Formal analysis, Investigation, Resources, Data curation, Writing – original draft, Visualization.

## Declaration of competing interest

The authors declare no conflicts of interest to report regarding the present study.

## Appendix Supplementary materials

**Supplementary Figure 1.** FKBP10 overexpression in breast cancer cell lines. (A–B) FKBP10 mRNA and protein levels in MCF7 and T47D cells after FKBP10 overexpression, as determined by RT-qPCR and WB. (C–D) Effects of FKBP10 over-expression on colony-forming ability and cell viability in MCF7 and T47D cells. Image, image 1 dummy alt text
