# Supplementary figures and images for: Pan-cancer analysis identifies FKBP10 as a regulator of tumor immunosuppression and therapeutic response
Source: Transl Oncol. 2026 Apr 3;67:102749. doi: 10.1016/j.tranon.2026.102749 (PMC13089051; doi:10.1016/j.tranon.2026.102749)

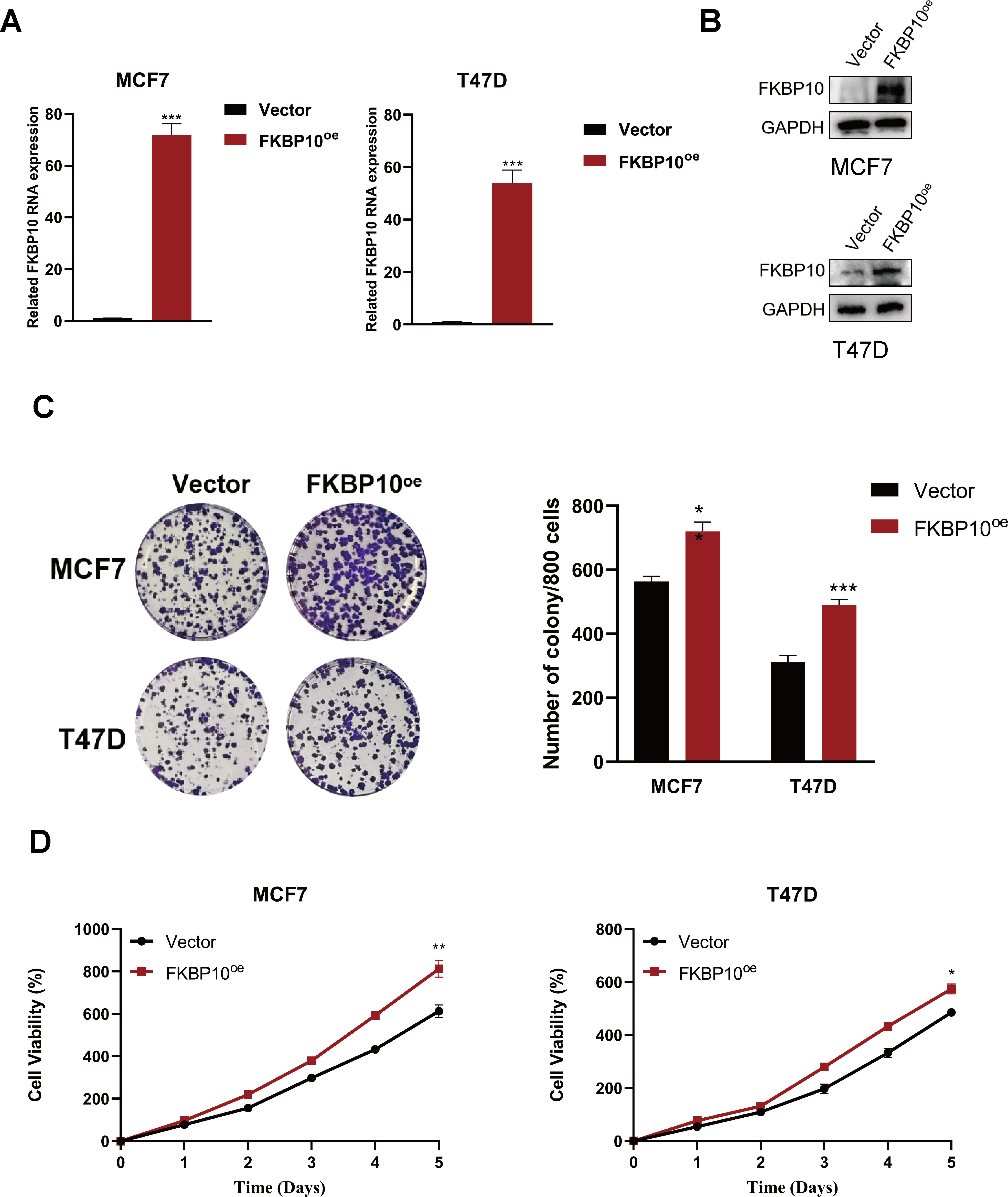

Supplement: Supplementary file 1 — Supplementary Figure 1. FKBP10 overexpression in breast cancer cell lines. (A–B) FKBP10 mRNA and protein levels in MCF7 and T47D cells after FKBP10 overexpression, as determined by RT-qPCR and WB. (C–D) Effects of FKBP10 over-expression on colony-forming ability and cell viability in MCF7 and T47D cells. [file mmc1.jpg]
